# DNA Damage Repair in Glioblastoma: A Novel Approach to Combat Drug Resistance

**DOI:** 10.1111/cpr.13815

**Published:** 2025-01-27

**Authors:** Ludovica Gaiaschi, Claudio Casali, Andrea Stabile, Sharon D'Amico, Mauro Ravera, Elisabetta Gabano, Andrea Galluzzo, Cristina Favaron, Federica Gola, Fabrizio De Luca, Serena Pellegatta, Maria Grazia Bottone

**Affiliations:** ^1^ Department of Biology and Biotechnology University of Pavia Pavia Italy; ^2^ Department of Sciences and Technological Innovation (DiSIT) University of Piemonte Orientale “A. Avogadro” Alessandria Italy; ^3^ Department of Sustainable Development and Ecological Transition University of Piemonte Orientale Vercelli Italy; ^4^ Unit of Immunotherapy of Brain Tumors Fondazione IRCCS Istituto Neurologico “C. Besta” Milan Italy

**Keywords:** chemotherapy, DNA damage response, drug resistance, glioblastoma

## Abstract

Due to the lack of effective therapeutic approach, glioblastoma (GBM) remains one of the most malignant brain tumour. By in vitro investigations on primary GBM stem cells, we highlighted one of the underlying mechanisms of drug resistance to alkylating agents, the DNA damage responses. Here, flow cytometric analysis and viability and repopulation assays were used to assess the long‐term cytotoxic effect induced by the administration of a fourth‐generation platinum prodrug, the (*OC*‐6‐44)‐acetatodiamminedichlorido(2‐(2‐propynyl)octanoato) platinum(IV) named Pt(IV)Ac‐POA, in comparison to the most widely used Cisplatin. The immunofluorescence studies revealed changing pathways involved in the DNA damage response mechanisms in response to the two chemotherapies, suggesting in particular the role of Poly (ADP‐Ribose) polymerases in the onset of resistance to Cisplatin‐induced cytotoxicity. Thus, this research provides a proof of concept for how the use of a prodrug which allows the co‐administration of Cisplatin and an Histone DeACetylase inhibitors, could suppress DNA repair mechanisms, suggesting a novel effective approach in GBM treatment.

## Graphical abstract

- Glioblastoma (GBM) stem cells are useful model for in vitro drug characterisation.
- DNA repair mechanisms can contribute to cancer cell survival.
- Pt(IV) bearing Histone DeACetylase inhibitors enhances anticancer effects by disrupting DNA damage response.
- Poly ADP ribose polymerases inhibitors could adjuvate GBM treatment.

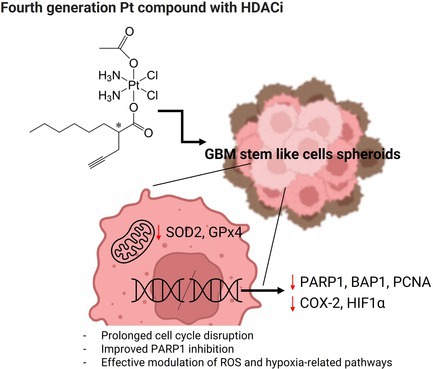

## 1 Introduction

The 2021 World Health Organisation (WHO) classification of Tumours of the Central Nervous System has redefined the molecular classification of these cancers, in particular, only Isocitrate DeHydrogenase‐1 wild‐type malignancies have been included under the name of glioblastoma (GBM), an adult‐type diffuse glioma. In this latest edition, the one thing that has not changed is the grading of GBM, which still remained a WHO grade IV tumour; therefore, a highly malignant tumour that due to the absence of effective therapy, leads to death in short periods of time [1].

Despite the need to classify a tumour in its entirety, the great molecular variability that can be recognised within a GBM mass remains evident; this and the presence of a heterogeneous population of cancer stem cells (CSCs) lead the bases to resistance to treatment and tumour recurrence [2]. To date, the therapies in use for the treatment of GBM are radiotherapy and chemotherapy, but despite those, it remains the most aggressive and deadly cancer. Hence, the need to identify therapeutic strategies that can reduce the self‐renewing and tumourigenic potential of glioblastoma stem cells (GSCs) in order to slow down GSC tumour growth and increase the overall patient survival.

Indeed, GSCs contribute to tumour malignancy through their sustained proliferation, invasiveness, stimulation of angiogenesis, suppression of antitumour immune responses, and resistance to cytotoxic therapies. Resistance may emerge due to alterations of protein expression levels in response to the used therapy, consequently leading to poor prognosis and relapses [3]. Thus, isolating and characterising GSCs could define new molecular targets for cancer therapy and determine novel roles for established signalling pathways in CSC biology. Here, to this purpose, we used primary GSCs enriched from Cavitron Ultrasonic Surgery Aspiration bags [4], subjecting them to Cisplatin and a fourth‐generation platinum prodrug, a complex named (*OC*‐6‐44)‐acetatodiamminedichlorido(2‐(2‐propynyl)octanoato) platinum(IV) (Pt(IV)Ac‐POA), to deepen the altered molecular mechanisms involved in the DNA damage response (DDR) [5] that could lead to Cisplatin resistance in GBM, and to identify new therapeutic targets to face the challenge of resistance. The Pt(IV)Ac‐POA allows the simultaneous co‐administration of Cisplatin and an Histone DeACetylase inhibitors (HDACi), that by increasing histones acetylation allows chromatin relaxing and modulation of the expression of DNA‐repair proteins, further increasing the persistence and efficacy of the Pt‐DNA adduct [6], indeed, the synergism of HDACi and cisplatin on neuroblastoma and other solid cancers has been proved [7, 8, 9, 10, 11].

As a matter of fact, it has been reported that if DNA repair pathways play a pivotal role in the maintenance of genome stability and integrity contributing to a limitation in carcinogenesis, they also can negatively impact the therapeutic efficacy of drugs primarily designed to induce cell death by direct or indirect DNA damage [12]. For example, the protein O6‐methylguanine‐DNA methyltransferase, which repairs O6‐methylguanine lesions removing the O6‐methyl group of guanine thus allowing double‐strand breaks repair mechanism, is known to be the main director of the resistance of GBM to temozolomide (TMZ) [13]. Moreover, recently, the role of RAD18 in the mechanisms of resistance to TMZ in glioma cells and the effect that HDACi may have in the downregulation of this protein has also been highlighted [14] and, analogously, various research focusing in other cancers indicated the impact of DDR, and in particular the contribution of the Poly ADP ribose polymerases (PARPs), proteins involved in the single‐strand breaks repair mechanism, in resistance to Cisplatin [15, 16, 17, 18, 19, 20].

Undeniably, Cisplatin is one of the most effective anticancer drugs used for a wide spectrum of solid tumours thanks to its cytotoxic effect on DNA, yet the results on gliomas are unsatisfactory, mainly for resistance induction [21, 22]. For this reason, targeting DNA repair in GBM is attracting new interest [19, 23, 24]. In other works, the positive effect of platinum complexes characterised by the presence of ligands with inhibitory HDACi activity on pancreatic and colon carcinoma cells, ovarian cancer and melanoma has been demonstrated [25, 26, 27], and the potential positive effect of HDACi on GBM cells has been shown [28, 29, 30, 31]. This work presents an innovative strategy by exploring a compound that holds the potential to deliver a dual therapeutic effect, leveraging the synergistic interaction between cisplatin and HDACi. The combinatorial effect of the two moieties characterising Pt(IV)Ac‐POA targets complementary pathways to overcome common limitations associated with separate drug administration. This combination aims to significantly enhance the therapeutic efficacy against GBM, offering a promising approach to overcome the challenges of current treatment modalities.

## 2 Materials and Methods

### 2.1 Cell Culture and Treatment for GSCs Lines

Immortalised human astrocytes were obtained from Professor Paolillo (Department of Drug Sciences, University of Pavia) and maintained in Dulbecco Modified Eagle Medium (DMEM)/F‐12 (Thermo Fisher Scientific) containing 10% DMEM (Euroclone), 10% heat‐inactivated foetal bovine serum (Euroclone), 1% L‐Glutamin (Euroclone), and 1X penicillin/streptomycin (Thermo Fisher Scientific) in T25 flask with vent cap, in a humidified incubator at 37°C and 5% CO_2_. Two different GSC enriched lines, obtained from the Department of Neurosurgery of Istituto Neurologico Carlo Besta (Milan, Italy) according to the protocol approved by the institutional Ethical Committee and already described [32], were evaluated (Table 1).

**TABLE 1:** Information regarding the patients and the respective tumours from which the primary cell cultures were isolated.

					Copy numbers variation							
Cells	Overall survival (months)	Age (years)	Gender	Tumoural subtype	EGFR	PDGFRA	HGFR	CDKN2a	PTEN	TP53	IDH1	MGMT
*BT487*	24	72	Male	Mesenchymal	+	+	+	wt	−	wt	wt	Unmethylated
*BT517*	8	48	Male	Classical	+	wt	+	−/−	−/−	c.524G>A	wt	Unmethylated

Abbreviations: CDKN2a, cyclin‐dependent kinase inhibitor 2a; EGFR, epidermal growth factor receptor; HGFR, hepatocyte growth factor receptor; IDH1, Isocitrate DeHydrogenase 1; MGMT, O‐6 MethylGuanine‐DNA MethylTransferase; PDGFRα, platelet‐derived growth factor receptor α; PTEN, Phosphatase and TENsin homologue; TP53, tumour protein 53; wt, wild type.

Human GBM spheres were cultured in DMEM/F‐12 GlutaMAX (Thermo Fisher Scientific) containing mitogenic factors 20 pg/μL hEGF and bEGF, 1X B27 supplement (Thermo Fisher Scientific), 1X penicillin/streptomycin (Thermo Fisher Scientific), and 1 μg/μL amphotericin B (EuroClone) and grown in suspension in T25 flask with vent cap, in a humidified incubator at 37°C and 5% CO_2_. For the following experiments, spheres were centrifuged at 300 rpm for 3 min and dissociated mechanically, dissociated cells were seeded in 96 multi‐well plates, as 4 × 10^4^ cells for each well, and upon spheres formation subjected to the treatments.

### 2.2 MTT

The cell viability test was conducted using the MTT salt (CAS 298‐93‐1, Calbiochem). Single cells were plated at density of 4 × 10^4^ cells/well in a 96‐well plate, after 48 h, tumour spheroids and astrocytes were subjected to the treatments. As a control, cells were incubated with the fresh culture medium and vehicle. Concentration ranges of 0–60 μM and 0–15 μM were tested for Cisplatin and Pt(IV)Ac‐POA, respectively, and doses of 0–800 μM for TMZ. After 48 h of exposure, the culture medium was replaced with fresh medium containing 1:10 of 5 mg/mL MTT solution in sterile PBS 1X (EuroClone). At the end of the 3 h incubation at 37°C, the precipitated MTT salts were dissolved in 100 μL DMSO/well. The samples' absorbance was measured using the Elx808TM Absorbance Microplate Reader (Bio‐Tek Instruments Inc.) at 490 nm.

### 2.3 Flow Cytometry

GSCs spheroids were treated in 25 cm^2^ flasks with Cisplatin or Pt(IV)Ac‐POA for 48 h of continued exposure at 37°C in a 5% CO_2_ humidified atmosphere. After treatments, cells were collected, washed in sterile PBS, dissociated and filtered with 40 μm cell strainers.

The cells were permeabilized in 70% ethanol for 10 min, treated with RNase A 100 U/mL, and stained for 10 min at room temperature with propidium iodide 50 μg/mL (Sigma‐Aldrich, Milan, Italy) 1 h. The preparation was processed with a Partec PAS III flow cytometer (Münster, Germany), PI red fluorescence was detected with a 610‐nm long‐pass emission filter. Data were analysed with Flowing Software 2.5.1.

### 2.4 Trypan Blue‐Exclusion Method

GSCs single cells were plated at density of 4 × 10^4^ cells/well in a 96‐well plate, after 48 h (time which allows spheroids formation), cells were treated as already indicated; after 48 h of continuous exposure at the different therapies, the cells were incubated with clean completed medium for 7 days. Cell viability was evaluated at three different time points: after 6 h of exposure to the therapy (t1), after 48 h of treatment (t2), and after the end of the recovery period (t3). Cells were collected, centrifuged at 300 rpm for 3 min, spheres were dissociated in 1 mL of PBS and 5 μL of suspension was mixed with 5 μL of 0.2% Trypan blue to check the cell viability. The count was effectuated with Bürker counting chamber.

### 2.5 Recovery Assay

This assay evaluated the ability of the cells under examination to reassociate to form spheroids at different time points after dissociation, reseeding, and treatment counting the remaining spheres and classifying them based on size. Then, 4 × 10^4^ cells/well were plated in a 96‐well plate as single cells. Then, 48 h after, spheres were moved in a 24‐well plate and treated; sphere were counted and classified after 6 and 48 h. After 48 h of acute treatment, spheres were dissociated and cells reseeded in fresh medium that was periodically changed for 7 days. At the end of the seventh day, spheres were counted, dissociated, and the medium was changed, and 96 h later, spheres and cells were counted once again. Spheres with a diameter < 100 μm had been classified as “small,” between 100 and 150 μm as “medium,” and above 150 μm as “large.”

### 2.6 Immunofluorescence Reactions

After treatments, floating spheres were collected from the supernatant and fixed in 4% formalin + Triton 1% (Sigma‐Aldrich, Milan, Italy) for 3 h at 4°C, the pellet was then resuspended in PBS 1X and moved to 0.5 mL tubes and conserved at 4°C for processing within 14 days.

Samples were rehydrated in PBS‐Triton 0.1% and washed in PBS‐Triton 0.1%‐BSA 3% as blocking for 1 h. Spheres were incubated over night with the primary antibodies diluted in PBS‐Triton 0.1% as in Table 2 at 4°C, washed three times in PBS‐Triton 0.1%, and incubated over night with secondary antibodies (Alexa 594 or 488 conjugated anti‐mouse or antirabbit antibody, Alexa Fluor, Molecular Probes, Invitrogen) diluted 1:500 in PBS‐Triton 0.1%. Then, 0.1 μg/mL of Hoechst 33,258 was added to counterstained the nuclei for 1 h. Spheres were washed three times in PBS‐Triton 0.1% and prepared for imaging in the next 48 h A Leica TCS SP8 STED 3X was used, and images were processed with LAS X software platform for all Leica microscopes. ImageJ Program 1.51 (NIH, MA, USA) was used to measure fluorescence intensity for semiquantitative analysis of protein expression.

**TABLE 2:** Primary antibodies used for immunofluorescence reactions.

Antigen	Primary antibody	Dilution
*PCNA*	Mouse monoclonal [PC10] anti‐PCNA (Abcam, Cambridge, UK)	1:200
*PARP‐1*	Rabbit polyclonal anti‐PARP‐1 (Cell Signalling Technology, Danvers, USA)	1:200
*BAP‐1*	Mouse monoclonal anti‐BAP1 (Santa Cruz Biotechnology, Dallas, USA)	1:200
*HIF‐1α*	Rabbit monoclonal anti‐HIF‐1α (D1S7W) (Cell Signalling Technology, Danvers, USA)	1:200
*AIF*	Rabbit polyclonal anti‐AIF (Cell Signalling Technology, Danvers, USA)	1:200
*COX‐2*	Mouse polyclonal anti‐COX2 (M‐19) (Santa Cruz Biotechnology, Dallas, USA)	1:200
*SOD‐2*	Rabbit monoclonal anti‐SOD2 (Cell Signalling Technology, Danvers, USA)	1:200
*GPx‐4*	Rabbit polyclonal anti‐GPx4 (Abcam, Cambridge, UK)	1:200

### 2.7 Statistical Analyses

Analyses of mean value ± SEM were done using one‐way ANOVA with Tukey test (GraphPad Prism 5.01). *p*‐Values from *p* < 0.05 were considered statistically significant.

## 3 Results

### 3.1 MTT

**FIGURE 1:**
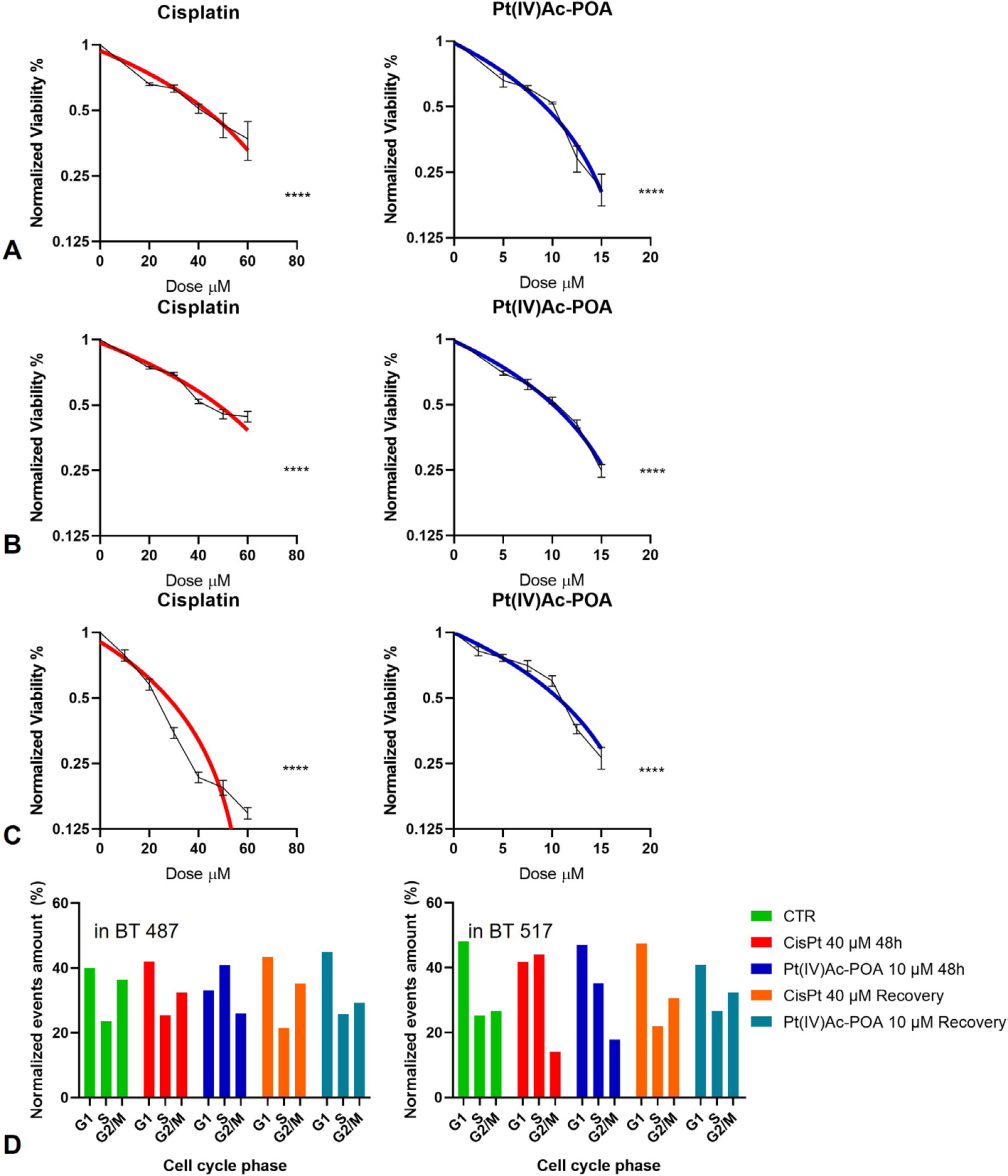
Curve representing viability of human (A) BT 487 and (B) BT 517 spheroids, and (C) astrocytes obtained using MTT assay after standard acute exposure, that is, 48 h continuous treatment, to increasing Cisplatin (0–60 μM) or Pt(IV)Ac‐POA (0–15 μM) concentrations. The relative cell viability is expressed as a percentage relative to the untreated control cells. Data representing the mean value ± SEM, statistical significance as **** for *p* value < 0.0001. (D) Histograms representing the distributions of GSCs cells, BT 487 on the left and BT 517 on the right, in the cellular cycle phases after each treatment at 48 h and after 7 days from the wash out.

To decide the optimal concentration of treatments to use for further analysis on the cell lines of interest, a viability MTT test (3‐(4,5‐dimethylthiazol‐2‐yl)‐2,5‐diphenyltetrazolium bromide) was performed on floating spheres or adhesive astrocytes. The range of concentration values evaluated were from 0 to 60 μM for Cisplatin and from 0 to 15 μM for Pt(IV)Ac‐POA. At 48 h of acute exposure to the drugs, the response of the two tumour cell lines were consistent. In Figure 1A–C, the statistically significance (*p* value < 0.0001) related to data obtained are reported. The IC50 (half‐maximal inhibitory concentration) on the GSCs' cell viability for the Cisplatin corresponded to 40 μM, meanwhile, for Pt(IV)Ac‐POA it was of 10 μM. Indeed, at the indicated concentration the percentage of viable BT 487 cells was 51.0% ± 5.1% and 52.6% ± 1.3% respectively, and of viable BT 517 cells was 51.9% ± 2.1% and 52.3% ± 3.1%, respectively. At the chosen concentrations for subsequent experiments, the astocytic culture showed a viability of 21.6% ± 2.1% when exposed to Cisplatin (IC50 30 μM) and 59.9% ± 5.8% when exposed to Pt(IV)Ac‐POA (IC50 12 μM). In addition, in Supplementary Figure 1, results on the viability of human BT 487 and BT 517 spheroids, and astrocytes obtained after 48 h continuous treatment to increasing TMZ (0–800 μM) concentration are reported. At 100 μM the percentage of viable BT 487 was 50.1% ± 0.1% and 52.4% ± 1.1% for BT 517, while at that same dose the percentage of viable astrocytes was 59.3% ± 10.9% (IC50 200 μM).

### 3.2 Flow Cytometry

To obtain preliminary data on the effect of the chemotherapy on the cell cycle flow cytometric analysis of sample stained with propidium iodide was adopted (Figure 1D and Supplementary Figure 2A,B).

In the BT 487 cell line, cisplatin induced an S‐phase blockade, increasing the percentage of cells in the S phase by 8.16% compared to the control, while cells in the G2/M phase decreased by 10.69%. After a recovery period, the cell cycle showed partial normalisation: cells in the G1 phase increased by 8.41%, cells in the S phase decreased by 8.66%, and cells in the G2/M phase decreased by 3.64%, thus indicating that cisplatin causes only a temporary slowdown in the cell cycle. Similar results were observed in the BT 517 cell line, where there was a 47.25% reduction in cells in the G2/M phase, with a corresponding increase in the S phase population. Following the recovery period, however the cell cycle also showed signs of recovery (−1.44% in G1, −12.97% in S, +14.92% in G2/M compared to CTR).

The new generation drug gave more promising results on the first cell line, showing an important reduction of cells in the G2/M phase (−28.71%) after 48 h of acute treatment with Pt(IV)Ac‐POA, this block in the G1/S phase was maintained even after the wash out with a cell population in the G2/M phase 19.53% lower than the control sample. On the contrary, on the BT 517 p7 line, despite a slowdown in the cycle at 48 h of exposure with −32.95% of cells in the G2/M phase, following the recovery period a shift to the right emerged (−14.98% in G1, +5.69% in S, +21.71% in G2/M compared to CTR).

### 3.3 Trypan Blue‐Exclusion Method

**FIGURE 2:**
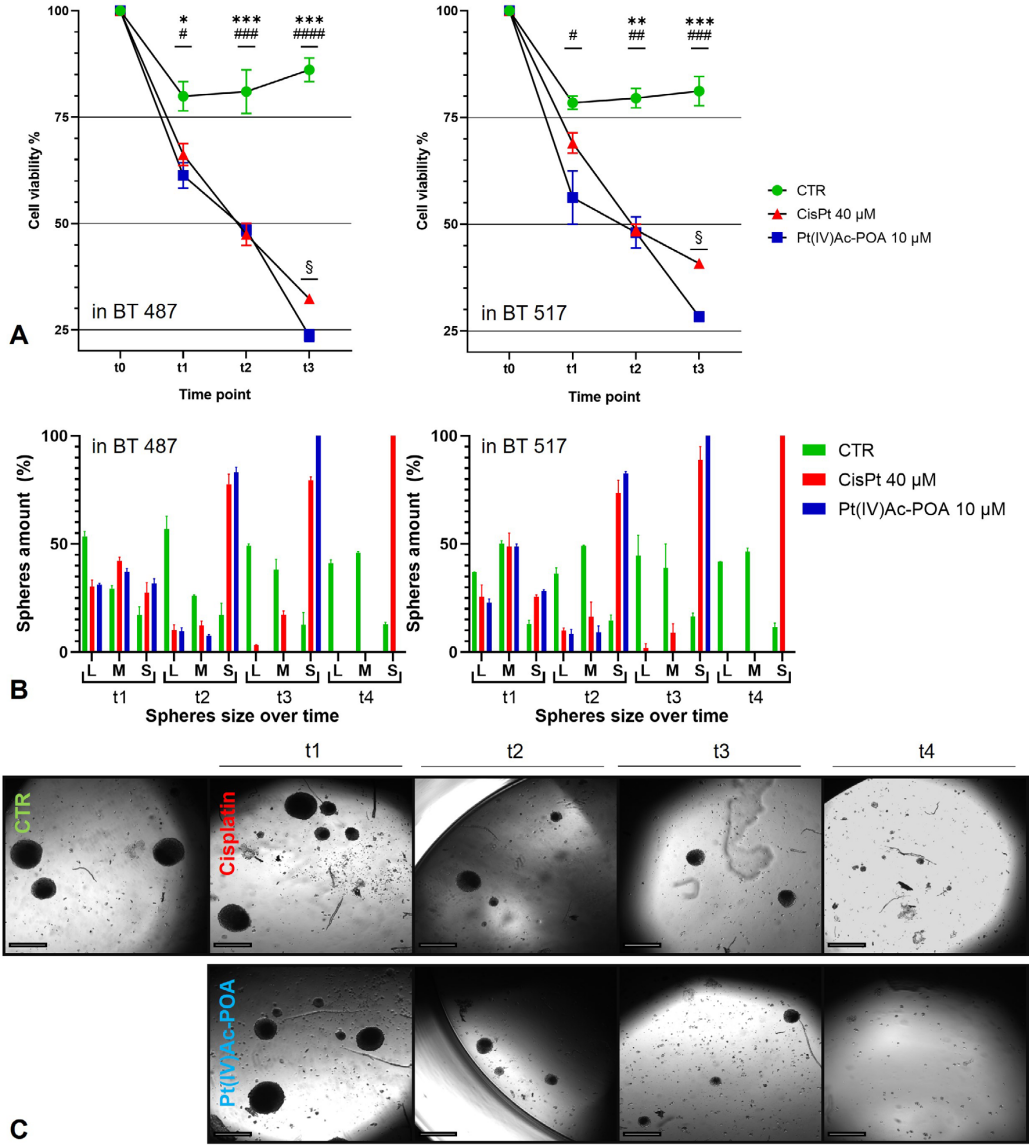
(A) Viability of GSCs cells, BT 487 on the left and BT 517 on the right, treated with Cisplatin 40 μM or Pt(IV)Ac‐POA 10 μM for 6 h (t1), 48 h (t2) and after 7 days of wash out, by Trypan blue exclusion test. Data representing the mean value ± SEM, statistical significance as: ****p < 0.0001, ***p < 0.001, **p < 0.01, *p < 0.1, specifically *CTR versus Cisplatin, #CTR versus Pt(IV)Ac‐POA, §Cisplatin versus Pt(IV)Ac‐POA. (B) Histograms representing the number of spheres classified by size after 6 h (t1) and 48 h (t2) from the beginning of the treatments with Cisplatin 40 μM or Pt(IV)Ac‐POA 10 μM, after 7 days from the wash out (t3) and after 96 h from the recovery period (t4). Data, from BT 487 on the left and BT 517 on the right, representing the mean value ± SEM. (C) Representative table of BT 487 cells, showing the resulting spheres after 6 h (t1) and 48 h (t2) from the beginning of the treatments with Cisplatin 40 μM or Pt(IV)Ac‐POA 10 μM, after 7 days from the wash out (t3) and after 96 h from the recovery period (t4). Magnification 4×, bar of 200 μm.

The different effects of the treatments in the cell lines analysed were also confirmed by a viability test (Figure 2A), exploiting the capability of a vital dye, such as Trypan blue, to cross compromised membranes.

If a decreasing trend in the cell viability of each species emerged already at 6 h of treatment, with a decrease compared to the control condition ranging from −12% to −17.16% after Cisplatin treatment and from −23.27% to −28.31% after the treatment with the fourth‐generation prodrug in the different lines, statistically significant results could be appreciated at 48 h of acute treatment. Line BT 487 saw a decrease of 41.45% ± 3.19% (*p*‐value = 0.0009) and 40.08% ± 1.82% (*p*‐value = 0.0007) following treatment with Cisplatin or Pt(IV)Ac‐POA, respectively. BT 517 cells, on the other hand, decreased the percentage of cell viability by −38.80% ± 1.65% (*p*‐value = 0.0026) and −39.55% ± 4.58% (*p*‐value = 0.0025), respectively. Following the recovery period of 7 days, the viability of BT 487 cells remained strongly impacted, a reduction of 62.50% ± 1.21% (*p*‐value = 0.0001) emerged thanks to exposure to Cisplatin and in particular with Pt(IV)Ac‐POA the decrease in vitality was equal to 72.58% ± 1.61% (*p* value < 0,0001), the different responses between the sample subjected to the two treatment appear significative with *p*‐value = 0.0392. The BT 517 line showed a reduced response to Cisplatin (−49.71% ± 1.03%, *p*‐value = 0.0007) but still promising to Pt(IV)Ac‐POA with a reduction of 65.09% ± 1.32% (*p*‐value = 0.0003), also in this case a significant difference emerged in the response to the two molecules with *p*‐value = 0.0199.

### 3.4 Recovery Assay

To provide a quantitative analysis of culture repopulation after drug treatment, the sphere forming capacity after Cisplatin 40 μM or Pt(IV)Ac‐POA 10 μM was evaluated, moreover whether surviving cells resume sphere formation after one‐week of recovery period and their self‐renewal capability after farther 96 h were observed (Figure 2B,C and Supplementary Figure 3) [33].

As shown in the graphs in Figure B, the chemotherapeutics used have induced over time the decrease of large and medium‐sized spheres in favour of smaller ones. On all the cellular specimens, the most promising results were obtained by administering Pt(IV)Ac‐POA, indeed, at t3 of treatment with Pt(IV)Ac‐POA only small spheres seemed to survive. Moreover, at t4, the fourth‐generation platinum compound impaired the formation of all‐size spheres.

There was an increase in the total number of spheres given in particular by an increase in smaller ones formation, in all the lines analysed at 48 h of acute treatments, the results were similar with both molecules in comparison to the control condition (Cisplatin +63.02% ± 9.81% or Pt(IV)Ac‐POA 66.04% ± 7.17 vs. Control condition considering the cell lines under examination). The trend described above, however, was reversed at subsequent time points. At 7 days after discontinuation of treatment, the percentage of spheres counted in the samples subjected to cisplatin therapy was +5.73% ± 6.68% compared to the control sample, while in the following 96 h it was −32.03% ± 1.25%, the reduction in the ability of tumour cells to organise themselves into spheroids was even more drastic following treatment with Pt(IV)Ac‐POA. The spheres counted after the recovery period were, in fact, only 12.60% ± 1.72% considering all the cell lines available compared to the untreated sample, up to 0 in the following 96 h.

### 3.5 Immunofluorescence Analysis

**FIGURE 3:**
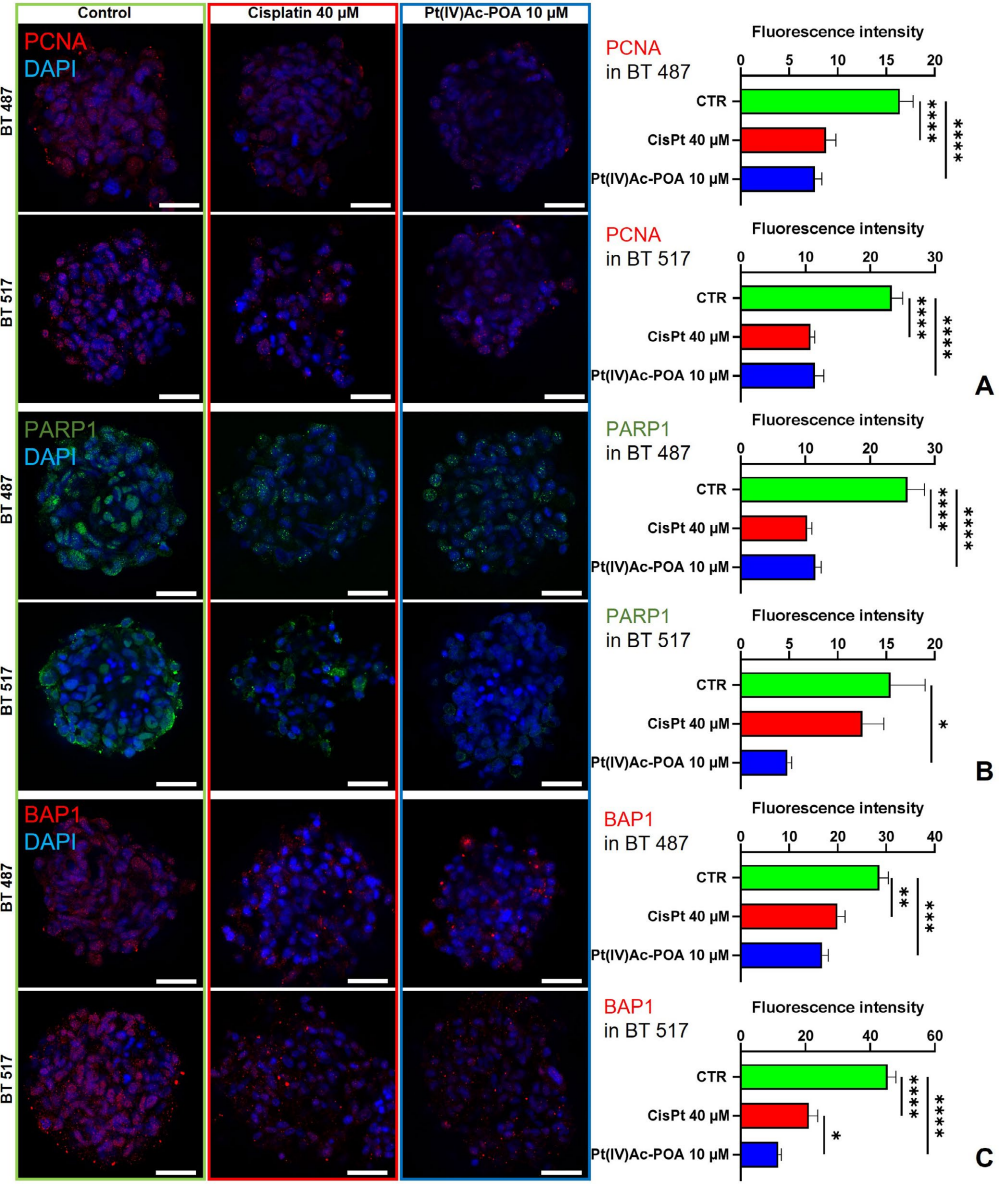
Proliferation and DNA repair activity. Immunolabelling for PCNA and PARP1 (in green) and BAP1 (in red): In the control condition and differently treated GSCs, that is, after 48 h‐CT Cisplatin 40 μM or Pt(IV)Ac‐POA 10 μM. DNA was stained with Hoechst 33,258 (blue fluorescence). Magnification 25×, bar of 40 μm. The histograms show the mean fluorescence intensity value of the immunolabelling ± SEM for (A) PCNA, (B) PARP1, and (C) BAP1. Statistical significance calculated as follows: ****p < 0.0001, ***p < 0.001, **p < 0.01, *p < 0.1.

**FIGURE 4:**
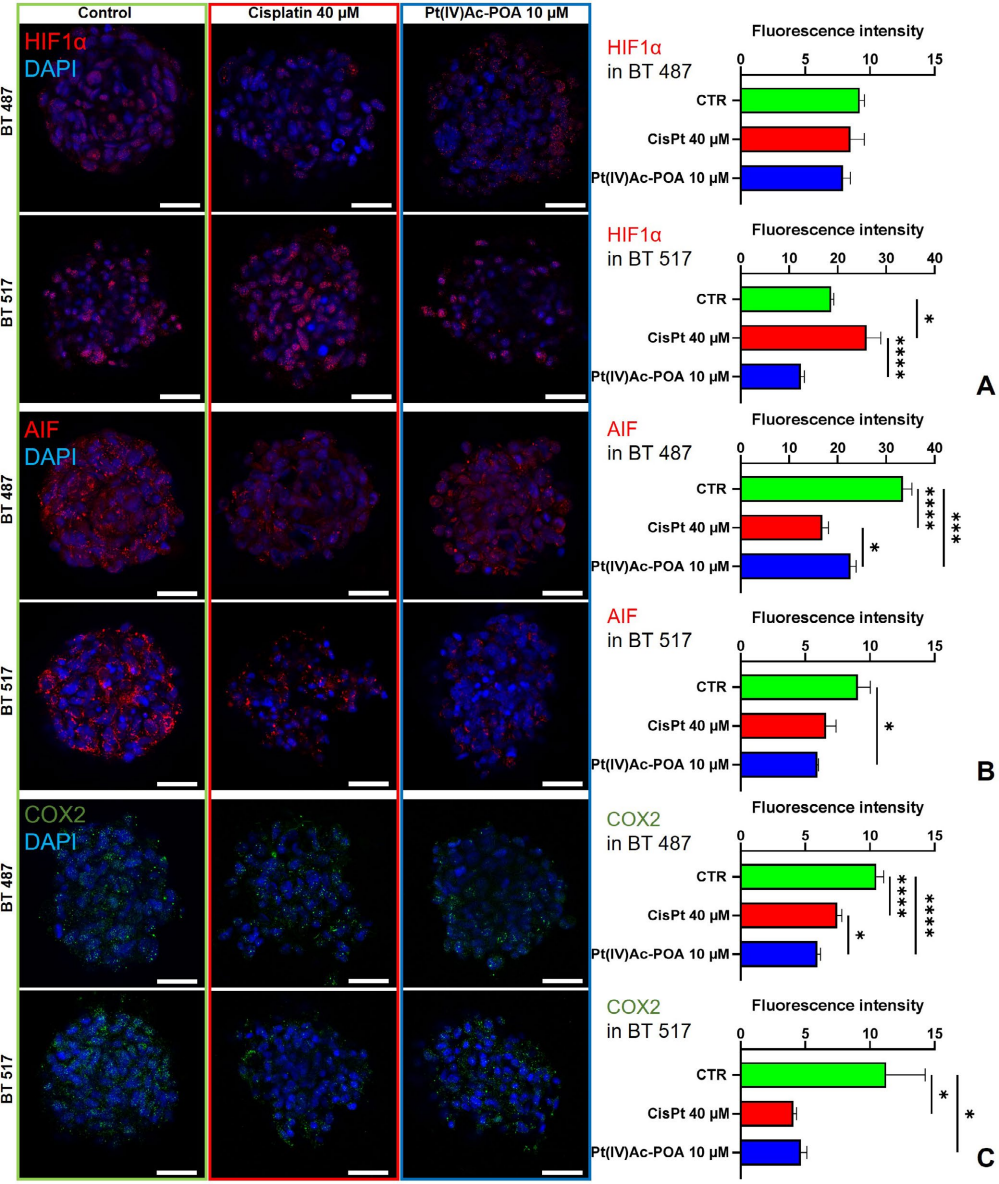
Hypoxic conditions, cell death and inflammatory response. Immunolabelling for HIF1α and AIF (in red) and COX2 (in green): In the control condition and differently treated GSCs, that is, after 48 h‐CT Cisplatin 40 μM or Pt(IV)Ac‐POA 10 μM. DNA was stained with Hoechst 33,258 (blue fluorescence). Magnification 25×, bar of 40 μm. The histograms show the mean fluorescence intensity value of the immunolabelling ± SEM for (A) HIF1α, (B) AIF, and (C) COX2. Statistical significance calculated as follows: ****p < 0.0001, ***p < 0.001, **p < 0.01, *p < 0.1.

**FIGURE 5:**
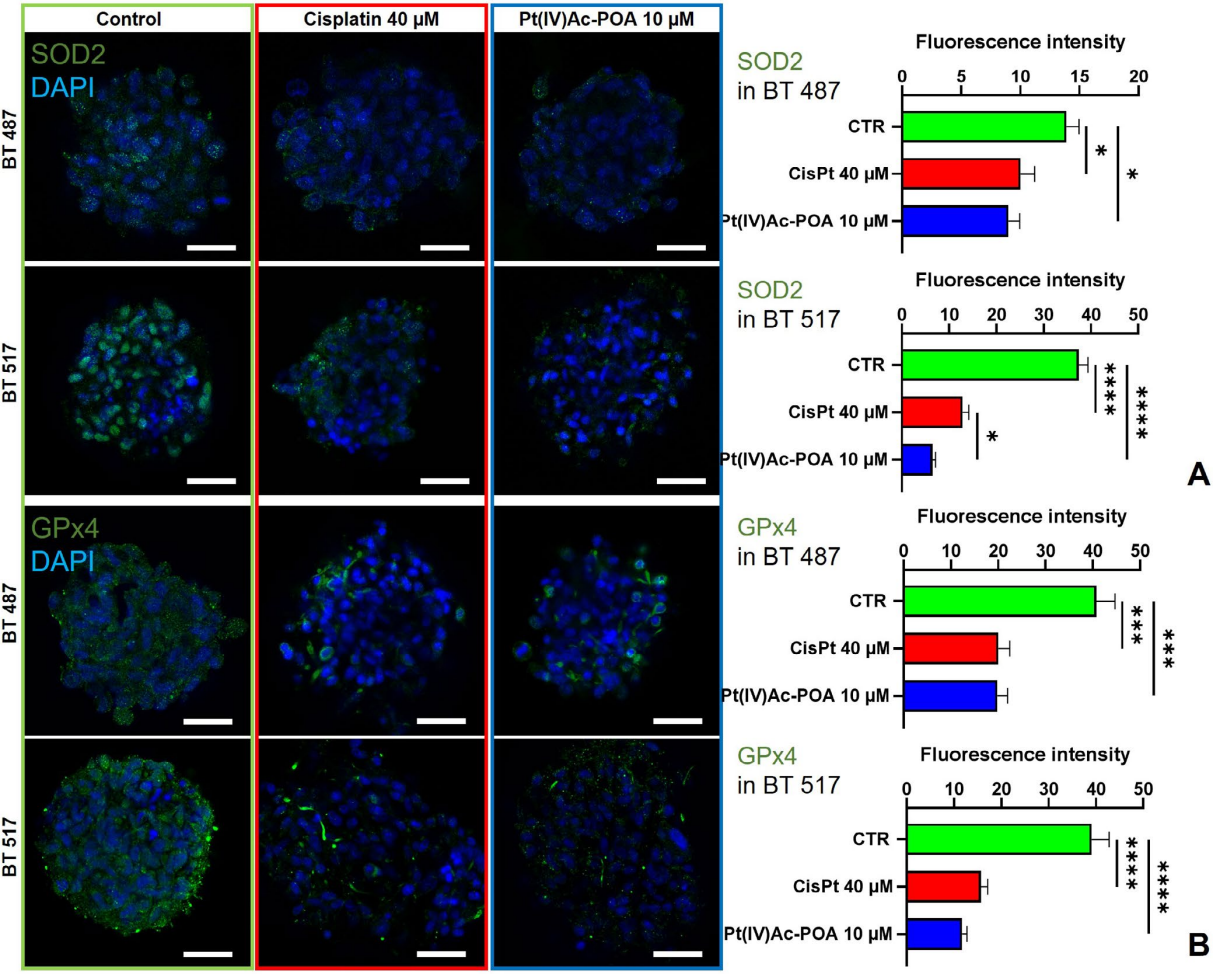
Oxidative stress. Immunolabelling for SOD2 and GPx4 (in green): In the control condition and differently treated GSCs, that is, after 48 h‐CT Cisplatin 40 μM or Pt(IV)Ac‐POA 10 μM. DNA was stained with Hoechst 33,258 (blue fluorescence). Magnification 25×, bar of 40 μm. The histograms show the mean fluorescence intensity value of the immunolabelling ± SEM for (A) SOD2 and (B) GPx4. Statistical significance calculated as follows: ****p < 0.0001, ***p < 0.001, **p < 0.01, *p < 0.1.

To provide insights into the cellular mechanisms related to DNA repair and cell proliferation (Figure 3) in response to different platinum compounds in GBM cells with different genomic landscapes, the expression of Proliferating Cell Nuclear Antigen (PCNA), PARP1 (in double immunofluorescence with apoptosis‐inducing factor [AIF], see Figure 4), and BAP1 (in double immunofluorescence with glutathione peroxidase 4 [GPX4], see Figure 5) was evaluated.

PCNA signalling, distributed as expected mainly in the nuclear compartment, showed a statistically significant reduction (*p*‐value = < 0.0001) compared to control samples after treatment with platinum‐based chemotherapeutics in both the analysed cell lines. The two specimens, which did not show any noticeable differences between them, after chemotherapy, also highlighted a decreased number of cells bearing PCNA immunolabelling. In details, the mean intensity of fluorescence measured was 16.42 ± 1.37 in the control sample of the BT 487 cells, that decreased of the 46.22% after the treatment with Cisplatin 40 μM for 48 h (8.83 ± 0.99) an of the 53.29% after the use of the fourth‐generation prodrug (7.67 ± 0.70). Similarly for the BT 517 cells, which were measured as 23.33 ± 1.71 of fluorescence intensity in the control sample, with a decrease, subsequent to the treatment with Cisplatin or Pt(IV)Ac‐POA of 53.71% or 50.84%, respectively (Cisplatin mean 10.80 ± 0.64, Pt(IV)Ac‐POA mean 11.47 ± 1.37). No statistical significative difference has been reported between the sample subjected to the two chemotherapies (Cisplatin vs. Pt(IV)Ac‐POA *p*‐value = 0.72 for BT 487 or *p*‐value = 0.93 for BT 517) (Figure 3A).

PARP1 signalling exhibited a reduction compared to control samples after treatment with platinum‐based chemotherapeutics, statistically significative difference (*p* value < 0.0001) emerged between the Cisplatin or Pt(IV)Ac‐POA‐treated samples in the BT 487 cell line; here a reduction of 60.03% or 55.31% occurred, respectively (CTR mean 25.80 ± 2.64 vs. Cisplatin mean 10.31 ± 0.69 or Pt(IV)Ac‐POA mean 11.53 ± 0.92), no noticeable difference between the response to the two treatments under analysis was highlighted (*p*‐value = 0.87). On the other hand, it emerged a decrease signal for PARP1 in the BT 517 line subjected to the only therapy with the new prodrug in comparison to both the control condition and the Cisplatin treated cells, of the 68.82% (*p* value < 0.0001) or 61.62% (*p*‐value = 0.061) (Pt(IV)Ac‐POA mean 4.82 ± 0.45 vs. CTR mean 15.46 ± 3.56 or Cisplatin mean 12.56 ± 2.20) (Figure 3B).

Consistently to the above‐mentioned markers, BAP1 signalling significantly decreased compared to control samples after treatment with platinum‐based chemotherapeutics. The signal revealed after the treatment with Cisplatin was reduced of 30.19% (*p*‐value = 0.0022) in the BT 487 and of 53.58% (*p* value < 0.0001) in the BT 517 (Cisplatin mean 19.98 ± 1.57 vs. CTR mean 28.62 ± 1.85 and Cisplatin mean 21.08 ± 2.75 vs. CTR mean 45.41 ± 2.54, respectively), moreover analysing the effect of the therapy with Pt(IV)Ac‐POA it emerged a reduction of 41.26% (*p*‐value = 0.0002) in the first cell line and of the 74.41% (*p* value < 0.0001) in the second one (Pt(IV)Ac‐POA mean 16.81 ± 1.26 or 11.62 ± 0.97, respectively). If no significant difference emerged between the two treatments in the BT 487 line (*p*‐value = 0.3929), in the BT 517 samples the reduction in the levels of BAP1 registered after the treatment with the fourth‐generation platinum compound was statistically relevant in comparison to the Cisplatin treated samples (*p*‐value = 0.0167) in particular considering the nucleus (*p*‐value = 0.0365) (Figure 3C).

Since the complexity and interconnection of multiple cellular pathways are well‐known, and it is recognised how components of DNA damage repair mechanisms can be influenced and, in turn, influence factors related to hypoxia or inflammation, correlatable markers have been considered (Figure 4).

From the analysis of HIF1a immunolabelling in the BT 487 line only a slight decrease in the signal, already weak in the non‐treated samples, in the sample subjected to chemotherapy emerged (CTR mean 9.22 ± 0.36 vs. Cisplatin mean 8.51 ± 1.06 *p*‐value = 0.7551 or Pt(IV)Ac‐POA 7.92 ± 0.57 *p*‐value = 0.3796). The corresponding analysis on the other cell line, BT 517, brought out a different trend, with a significant increased presence of HIF1a of the 39.15% in the samples exposed to Cisplatin in comparison to the untreated cells and a decreasing of the fluorescence intensity for the marker of the 33.53% in the Pt(IV)Ac‐POA‐treated ones (CTR mean 18.67 ± 0.54 vs. Cisplatin mean 25.98 ± 2.94 *p*‐value = 0.0202 or Pt(IV)Ac‐POA 12.91 ± 0.73 *p*‐value = 0.0504); interestingly, the difference revealed between the cells treated with Cisplatin or Pt(IV)Ac‐POA resulted statistically significant with *p* value < 0.0001 (Figure 4A).

AIF signalling showed a statistically significant reduction compared to control samples (CTR mean 33.49 ± 1.85 vs. Cisplatin mean 16.87 ± 1.29 or Pt(IV)Ac‐POA 22.69 ± 1.17) after treatment with Cisplatin or Pt(IV)Ac‐POA in BT 487 cell, of 49.63% (*p* value < 0.0001) or 32.25% (*p*‐value = 0.0002), respectively, noticeably the difference in the response to the two drugs was also significant with *p*‐value = 0.0304. Similarly, after the treatment with the chemotherapies a reduction in the amount of AIF was noticed in BT 517 cells, a decrease of the 27.09% or 34.69% was registered in the samples treated with Cisplatin or the new prodrug, respectively (CTR mean 9.08 ± 0.95 vs. Cisplatin mean 6.62 ± 0.75 *p*‐value = 0.0525 or Pt(IV)Ac‐POA 5.93 ± 0.09 *p*‐value = 0.0109). The more important difference was revealed consistently in the cytosolic compartment of the Pt(IV)Ac‐POA‐treated cells (Figure 4B).

In the cells under investigation COX2 signalling displayed a consistent reduction compared to control samples after treatment with the platinum compounds, the decrease was of the 28.95% or 43.43% after the treatment with Cisplatin or Pt(IV)Ac‐POA in BT 487 cell (CTR mean 10.50 ± 0.57 vs. Cisplatin mean 7.46 ± 0.37 *p* value < 0.0001 or Pt(IV)Ac‐POA 5.94 ± 0.25 *p* value < 0.0001), the response at the different drugs appeared significant with *p*‐value = 0.0425. In the BT 517, the reduction observed in the fluorescent intensity related to COX2 was of 63.68% in Cisplatin treated cells (*p*‐value = 0.0221), and of 58.53% in Pt(IV)Ac‐POA‐treated samples (*p*‐value = 0.0372) (CTR mean 11.26 ± 3.02 vs. Cisplatin mean 4.09 ± 0.24 or Pt(IV)Ac‐POA 4.67 ± 0.46) (Figure 4C).

Given the role of oxidative species in DNA damage mechanisms, an assessment of markers indicative of intracellular redox status was performed.

A decreasing in the amount of superoxide dismutase 2 (SOD2) emerged consistently in all the cell line subjected to the platinum‐based chemotherapy in particular the stronger effect was registered in the BT 517 cells. Indeed, the decrease measured in BT 487 cells was of 27.95% (*p*‐value = 0.0467) after Cisplatin treatment and of 35.27% (*p*‐value = 0.0108) after Pt(IV)Ac‐POA (CTR mean 13.92 ± 1.06 vs. Cisplatin mean 10.03 ± 1.20 or Pt(IV)Ac‐POA 9.01 ± 0.96); on the other hand, in BT 517 cells the decrease of the measured fluorescence intensity in the sample exposed to Cisplatin was of 65.74% (*p* value < 0.0001) and of 82.58% (*p* value < 0.0001) in those exposed to the new prodrug, revealing also a statistically significant difference between the sample subjected to the two chemotherapies (*p*‐value = 0.0152) (CTR mean 37.48 ± 1.92 vs. Cisplatin mean 12.84 ± 1.36 or Pt(IV)Ac‐POA 6.53 ± 0.59) (Figure 5A).

The quantification of the signal from GPx4 immunolabelling showed a consistent and significative reduction of the protein amount compared to control samples after treatment with the platinum compounds in all cell lines (BT 487 CTR mean 40.81 ± 3.92 vs. Cisplatin mean 20.06 ± 2.42 or Pt(IV)Ac‐POA 19.86 ± 2.18; BT 517 CCTR mean 39.12 ± 3.69 vs. Cisplatin mean 15.69 ± 1.45 or Pt(IV)Ac‐POA 11.72 ± 1.05). The sample exposed to Cisplatin showed a reduction of 50.85% or 59.89% (*p*‐value = 0.0001) in BT 487 or BT 517, respectively, while, those exposed to Pt(IV)Ac‐POA showed a mean fluorescence intensity 51.34% or 70.04% lower (*p* value < 0.0001) in the same lines, respectively (Figure 5B).

## 4 Discussion

GBM, a grade IV adult‐type diffuse glioma, is the most aggressive form of brain tumour and is notoriously resistant to treatment. Current therapies are insufficient to overcome drug resistance. Understanding the behaviour of GSCs in response to different treatments could reveal new therapeutic targets. This study compares the effects on DNA damage and hypoxia signalling of Cisplatin, a widely used anticancer drug, with its fourth‐generation derivative, Pt(IV)Ac‐POA, which includes an HDACi as an axial ligand, on two primary GSC lines. In previous studies, the high efficacy of the fourth‐generation compound on established human and rat GBM and neuroblastoma cell lines [34, 35, 36, 37, 38], along with its superior intracellular accumulation capacity [6], has been demonstrated. These promising results have further motivated the evaluation of the compound in more complex models, such as 3D spheroids of stem‐like cells.

Here, it was shown that both drugs were effective in reducing cell proliferation, with a cytotoxic activities on GSCs superior to the one of TMZ, but their long‐term impacts varied. Key findings indicate that Cisplatin (40 μM) disrupted the cell cycle significantly after 48 h of continuous treatment, but this effect was not sustained once the treatment ceased, as cells reverted to normal cycling. In contrast, Pt(IV)Ac‐POA (10 μM) caused a prolonged block in the G1/S phase, especially in the BT 487 cell line. Continuous exposure (6 or 48 h) followed by a 7‐day recovery period showed that Pt(IV)Ac‐POA maintained stronger long‐term antitumour activity compared to Cisplatin. Furthermore, tests focusing on cell survival and spheroid reformation after treatment and dissociation confirmed the enduring impact of Pt(IV)Ac‐POA.

The study of PCNA levels, involved in DNA replication, cell division, DNA repair and also cytosolic metabolic activities, that is, glycolysis, pentose phosphate pathway, and Akt signalling, thus being associated with tumour aggressiveness [17], indicated that the promising effects on survival at 48 h of treatment were corroborated, highlighting the superior efficacy of the new platinum compound over Cisplatin.

Further evidence that Pt(IV)Ac‐POA better inhibits the cells' ability to respond to DNA damage emerged from the study of PARP1, an enzyme involved in the base excision repair pathway. Numerous studies have shown that targeting PARP 1 with specific inhibitors may improve prognosis for breast or ovarian cancer patients with mutated BRCA1/2 genes, which are involved in homologous recombination repair [39, 40]. Disrupting DDR processes in cancer cells aggravates genomic DNA damage, triggering senescence or programmed cell death [41]. Review studies have highlighted the impactful role of PARP inhibitors in various tumours [42]. In particular, studies on GBM patients have shown that Phosphatase and Tensin homologue (PTEN)‐deficient cells, such as one of the primary lines tested (BT 517), which showed higher levels of PARP1 after Cisplatin treatment, could benefit from combining PARP inhibitors with standard treatments [43]. Here, indeed, we determined the efficacy of Pt(IV)Ac‐POA in GBM cells, founding that it consistently reduce the amount of PARP1, even where Cisplatin treatment was ineffective. This suggests a role in the disruption of the DNA repair mechanisms by the fourth generation prodrug, which could lead to the accumulation of DNA damage.

We also investigated the response of BAP1 to the administration of Cisplatin and Pt(IV)Ac‐POA. BRCA1‐associated protein‐1, a deubiquitinating enzyme involved in DNA repair and chromatin organisation, typically acts as a tumour suppressor but shows abnormal behaviour in gliomas [44]. In glioma cells, BAP1 is localised in both the nucleus and cytoplasm. High cytoplasmic BAP1 is associated with low overall survival, while nuclear BAP1 abundance is not correlated with survival [45]. Our studies indicated that treatment with Pt(IV)Ac‐POA significantly decreased BAP1 levels in both the cytoplasm and nucleus, suggesting that Pt(IV)Ac‐POA effectively impairs DNA damage repair mechanisms, in fact, it has been proven that PARP1 activates and interacts with BAP1 [46, 47]. Additionally, reduced cytoplasmic BAP1 may increase GBM sensitivity to tumour necrosis factor, enhancing proapoptotic effects [48] and reducing the pro‐survival repression of the unfolded protein response [49]. This could contribute to endoplasmic reticulum (ER) stress‐induced cell death [50].

Mitochondria and the ER interact significantly during apoptotic events [51]. In response to ER stress, the AIF, which is normally localised in the mitochondria, translocates to the cytoplasm where it is cleaved. Research indicates that in advanced lung adenocarcinomas, suppressed AIF expression correlates with increased drug resistance via c‐Met [52], while high AIF expression in various tumours is linked to better patient survival [53, 54] and inhibition of hypoxia‐induced epithelial to mesenchymal transition [55]. Our analyses revealed that Pt(IV)Ac‐POA maintained higher levels of AIF in the BT 487 cell line, whereas results in other cell lines were less promising, suggesting possible mechanisms of resistance in GBM cells. AIF levels in tumours are inversely correlated with hypoxia, which inhibits AIF expression through the hypoxia‐inducible factor‐1α (HIF1α) [55]. HIF1α is crucial for adaptive responses to low oxygen levels, influencing processes like angiogenesis, metabolic reprogramming, invasiveness, and therapy resistance. Downregulation of HIF1/2α in hypoxia condition impairs cancer cell invasion and growth [56], and HIF1α inhibition in tumour‐associated macrophages can enhance antitumour immunity [57]. Our studies indicated that Pt(IV)Ac‐POA effectively maintains or reduces basal levels of HIF1α, even in tumour cells with resistance characteristics that otherwise show increased HIF1α in response to Cisplatin, suggesting that other pathways might be involved in the complex interplay between hypoxia, cell death, and drug resistance.

This result is particularly encouraging because it aligns with previous findings that over‐expressed HIF1α proteins are present in PTEN−/− disease models [58, 59] and GBM‐derived cell lines, where PTEN negatively regulates Akt activation of HIF1α activity [60]. Additionally, a connection has emerged linking Cisplatin resistance in PTEN−/− cell lines to HIF1α protein management and PARP1 inhibition [61, 62], which may also cause HIF1α post‐transcriptional instability [63]. The new generation compound Pt(IV)Ac‐POA is crucial in this context, as its HDACi axial ligand could potentially downregulate or inactivate PARP1 at both transcriptional and post‐translational levels [64, 65]. This is particularly relevant because tumours with p53 loss, such as the BT 517 line, show increased dependency on PARP‐associated repair pathways [66], highlighting the importance of Pt(IV)Ac‐POA in targeting these mechanisms.

Closely linked to hypoxia factors, Cyclooxygenase‐2 (COX‐2) is an enzyme involved in inflammation [67] and is strongly associated with malignant progression in GBM, selective COX‐2 inhibition has been shown to reduce proliferation and migration in GBM cell lines [68, 69, 70]. Given its role in glioma invasion, angiogenesis, and immunosuppression, COX‐2 inhibition can sensitise GBM to chemo‐ and radio‐therapies [71]. Our results indicate that new generation drugs, including Pt(IV)‐derivatives, are as effective as, or even more effective than, Cisplatin in reducing COX‐2 levels. This suggests that Pt(IV)‐derivatives could help overcome resistance mechanisms employed by tumours.

Another controversial topic in tumour biology is related to the role of reactive oxygen species (ROS) and free radicals, as increased ROS can both promote tumourigenesis and initiate oxidative stress‐induced programmed cell death [72, 73, 74]. ROS are also crucial in radiation‐induced DNA damage [75], making the balance between oxidative stress and antioxidant defences vital in cancer biology.

In this study, the administration of platinum compounds resulted in reduced levels of two key antioxidant enzymes: SOD2 and GPx4; both carried by the inner mitochondrial membrane, the first one converts superoxide radicals into hydrogen peroxide, which is reduced to water and oxygen by the latter [76]. GPx4 is also known as it inhibits lipid peroxidation [77]. A strong link between SOD2 and GPx4 activity has been highlighted in numerous studies, which have also demonstrated an interconnection between ROS, and in particular O^2−^, increased Fe^2+^, lipid peroxidation and therefore cell death by activation of the ferroptotic pathway, in which it seems to be also involved Akt/ SIRT3/SOD2 signalling [78, 79]. Although knowledge about SOD2 in brain tumours is limited, recent studies suggest that reduced SOD2 levels correlate with lower tumour proliferation and longer survival in TMZ‐resistant GBM models [80]. Additionally, it has been highlighted that GPx4 inhibition mediates ferroptosis [81], a form of regulated iron‐dependent cell death, disconnected from necroptosis or apoptosis, driven by toxic accumulation of lipid peroxides on cellular membranes [77]. Treatment with the fourth‐generation platinum compound effectively modulates both SOD2 and GPx4 and induces metabolic stress, suggesting that combining HDAC inhibitors with Cisplatin could reduce drug resistance and enhance therapeutic efficacy.

## 5 Conclusions

Despite significant progress, many aspects of GBM resistance mechanisms remain unclear, particularly why treatments like Cisplatin are less effective against these tumours compared to other solid cancers. The findings from this study underscore the crucial role of DNA damage repair mechanisms in enabling GBM to evade the cytotoxic effects of platinum‐based therapies. Inhibiting PARP activity emerges as a potent strategy for enhancing GBM treatment, especially in tumour types already deficient in other DNA repair pathways. The potential of fourth‐generation compounds, such as Pt(IV)Ac‐POA, which incorporate HDACi, to disrupt DNA repair, modulate hypoxia‐related factors, and alter antioxidant defences, highlights their promise. Pt(IV)Ac‐POA's ability to target PARP1 and its associated pathways offers a promising approach to overcoming drug resistance and improving therapeutic outcomes in GBM.

## Author Contributions

Conceptualisation: Ludovica Gaiaschi, Claudio Casali, Mauro Ravera, Serena Pellegatta, and Maria Grazia Bottone. Data curation: Ludovica Gaiaschi, Claudio Casali, Andrea Stabile, Sharon D'Amico, Andrea Galluzzo, Cristina Favaron, Federica Gola, and Fabrizio De Luca. Formal analysis: Ludovica Gaiaschi, Claudio Casali, Andrea Stabile, and Sharon D'Amico. Funding acquisition: Maria Grazia Bottone. Investigation: Ludovica Gaiaschi, Claudio Casali, Andrea Stabile, Sharon D'Amico, Andrea Galluzzo, Cristina Favaron, Federica Gola, and Fabrizio De Luca. Methodology, Ludovica Gaiaschi, Claudio Casali, Elisabetta Gabano, Mauro Ravera, Andrea Galluzzo, Serena Pellegatta, and Maria Grazia Bottone. Project administration: Serena Pellegatta and Maria Grazia Bottone. Resources: Mauro Ravera, Serena Pellegatta, and Maria Grazia Bottone. Supervision: Mauro Ravera, Serena Pellegatta, and Maria Grazia Bottone. Visualisation: Ludovica Gaiaschi, Claudio Casali, Andrea Stabile, and Sharon D'Amico. Writing – original draft: Ludovica Gaiaschi. Writing – review and editing: Claudio Casali, Mauro Ravera, Andrea Galluzzo, Serena Pellegatta, and Maria Grazia Bottone. All authors have read and agreed to the published version of the manuscript.

## Ethics Statement

The authors have nothing to report.

## Consent

The authors have nothing to report.

## Conflicts of Interest

The authors declare no conflicts of interest.

## Supporting information


**SUPPLEMENTARY FIGURE 1** Curve representing viability of human BT 487 and BT 517 spheroids, and astrocytes obtained using MTT assay after standard acute exposure, that is, 48 h continuous treatment, to increasing Temozolomide (0–800 μM) concentrations. The relative cell viability is expressed as a percentage relative to the untreated control cells. Data representing the mean value ± SEM.

**SUPPLEMENTARY FIGURE 2** Representative histograms of the cytofluorimetric analysis of (A) BT 487 and (B) BT 517 cell cycle status. Cytograms of the DNA content after IP staining, in control conditions, after treatment with Cisplatin 40 μM or Pt(IV)Ac‐POA 10 μM for 48 h and after 7 days of wash out.

**SUPPLEMENTARY FIGURE 3** Representative table of BT 517 cells, showing the resulting spheres after 6 h (t1) and 48 h (t2) from the beginning of the treatments with Cisplatin 40 μM or Pt(IV)Ac‐POA 10 μM, after 7 days from the wash out (t3) and after 96 h from the recovery period (t4). Magnification 4×, bar of 200 μm.

## Data Availability

Data sharing is not applicable to this article as no new data were created or analysed in this study.
