# Supplementary figures and images for: DNA Damage Repair in Glioblastoma: A Novel Approach to Combat Drug Resistance
Source: Cell Prolif. 2025 Jan 27;58(6):e13815. doi: 10.1111/cpr.13815 (PMC12179567; doi:10.1111/cpr.13815)

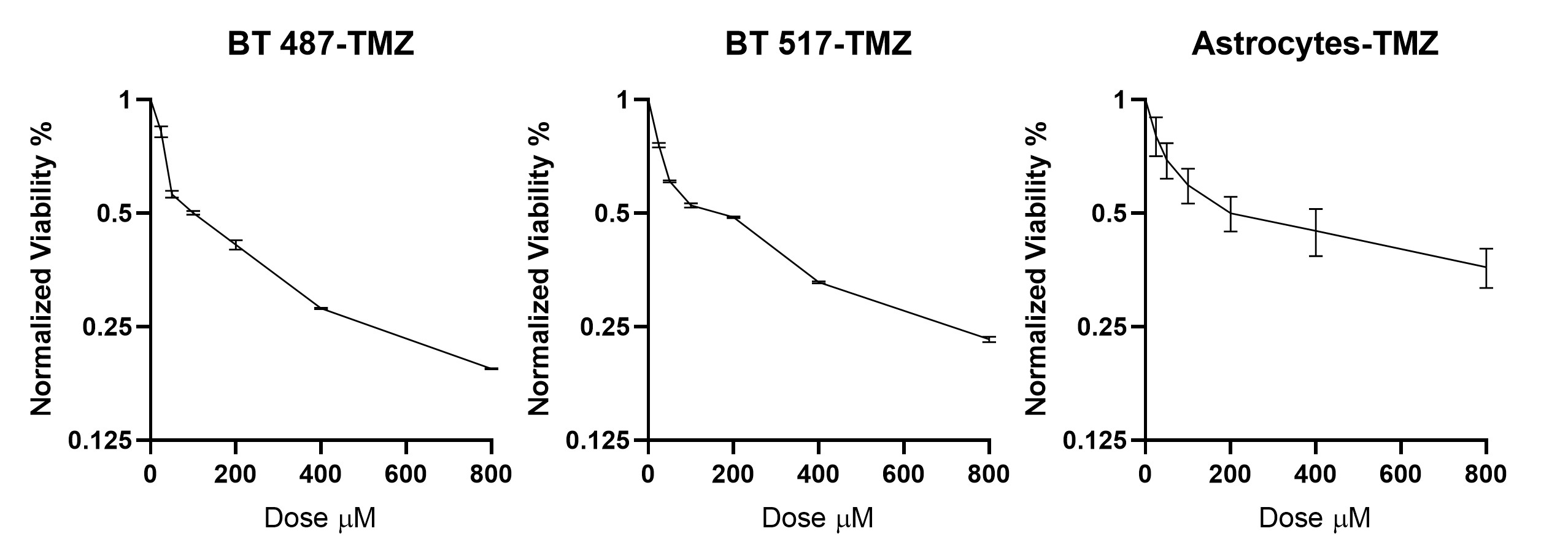

Supplement: Supplementary file 1 — SUPPLEMENTARY FIGURE 1 Curve representing viability of human BT 487 and BT 517 spheroids, and astrocytes obtained using MTT assay after standard acute exposure, that is, 48 h continuous treatment, to increasing Temozolomide (0–800 μM) concentrations. The relative cell viability is expressed as a percentage relative to the untreated control cells. Data representing the mean value ± SEM. [file CPR-58-e13815-s003.jpg]

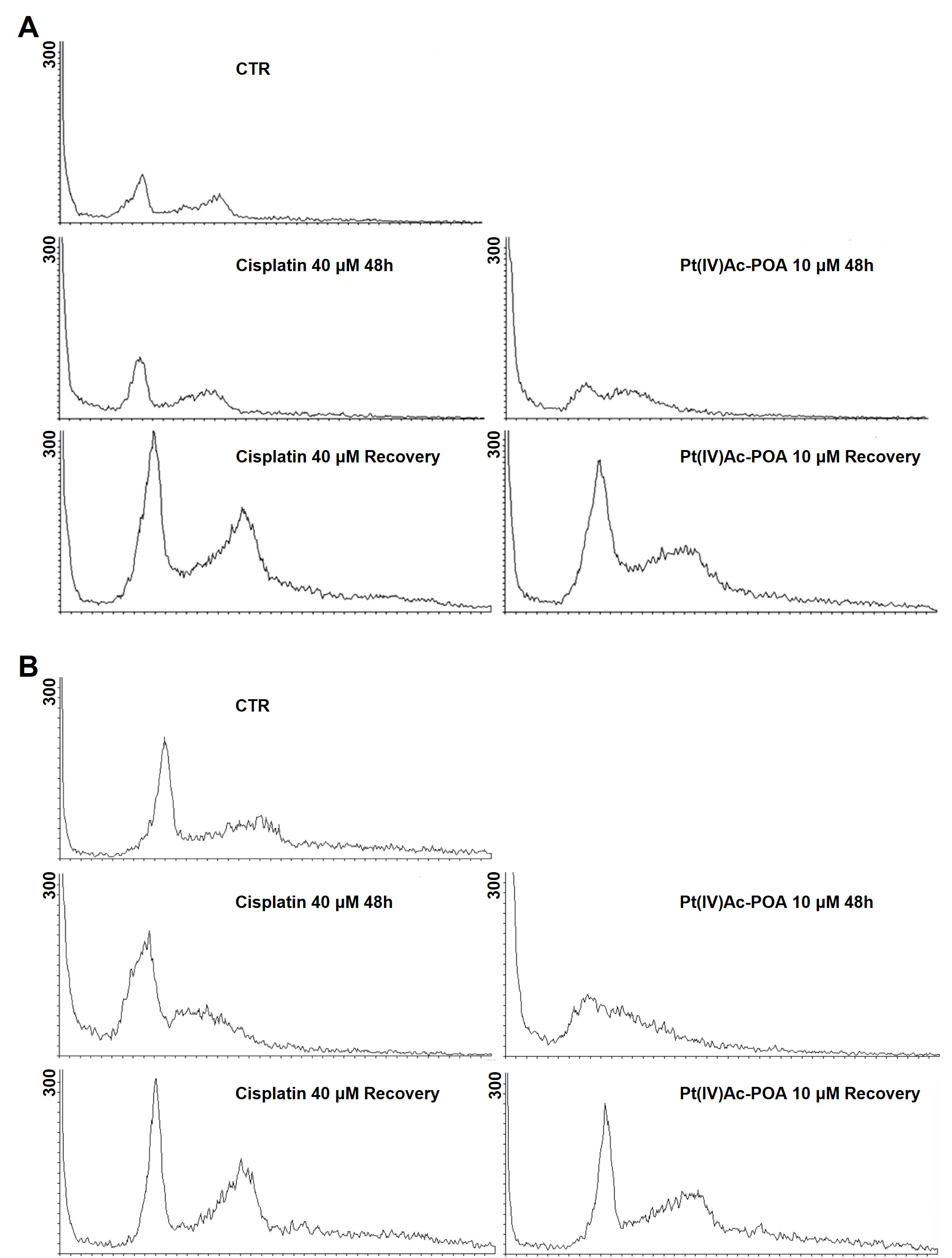

Supplement: Supplementary file 2 — SUPPLEMENTARY FIGURE 2 Representative histograms of the cytofluorimetric analysis of (A) BT 487 and (B) BT 517 cell cycle status. Cytograms of the DNA content after IP staining, in control conditions, after treatment with Cisplatin 40 μM or Pt(IV)Ac‐POA 10 μM for 48 h and after 7 days of wash out. [file CPR-58-e13815-s001.jpg]

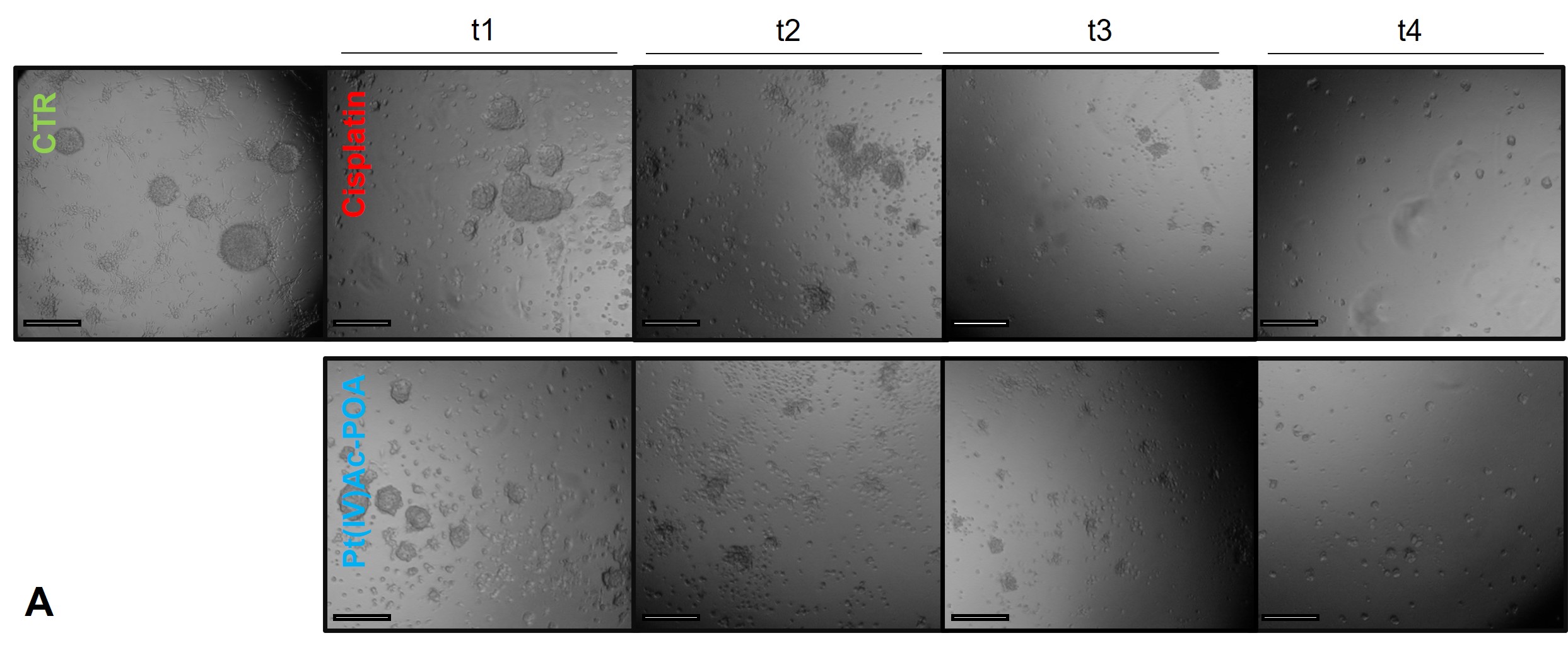

Supplement: Supplementary file 3 — SUPPLEMENTARY FIGURE 3 Representative table of BT 517 cells, showing the resulting spheres after 6 h (t1) and 48 h (t2) from the beginning of the treatments with Cisplatin 40 μM or Pt(IV)Ac‐POA 10 μM, after 7 days from the wash out (t3) and after 96 h from the recovery period (t4). Magnification 4×, bar of 200 μm. [file CPR-58-e13815-s002.jpg]
